# Species Diversity, Host Association, and Evolutionary History of *Cronartium*: An Important Global Fungal Pathogen to Trees

**DOI:** 10.1002/ece3.70545

**Published:** 2024-11-10

**Authors:** Jingyu Zhang, Clement K. M. Tsui, Chongjuan You

**Affiliations:** ^1^ Beijing Key Laboratory for Forest Pest Control, College of Forestry Beijing Forestry University Beijing China; ^2^ Infectious Disease Research Laboratory National Center for Infectious Diseases Singapore Singapore; ^3^ Lee Kong Chian School of Medicine Nanyang Technological University Singapore Singapore; ^4^ Faculty of Medicine University of British Columbia Vancouver Canada

**Keywords:** biogeography, divergence, genetic diversity, host association, *rust*

## Abstract

Pine stem rust, the most damaging and widespread forest disease occurring in pine trees in the Northern Hemisphere, is primarily caused by *Cronartium* species (Pucciniales, Melampsorineae). While the phylogenetic relationships of major *Cronartium* species have been largely elucidated, there is limited understanding of their species diversity and the evolutionary processes shaping their distribution patterns. In this work, we performed broad sampling and sequencing of *Cronartium* taxa in China together with additional sequence data and other accessions in NCBI to investigate the diversification and to estimate the divergence time of major evolutionary events in this genus. Molecular dating analysis suggested that the divergence of the genus *Cronartium* probably was around 91.78 Ma during the Upper Cretaceous. It is believed that *Cronartium* species may have originated in Asia and North America, with intercontinental dispersals occurring primarily during the Middle Eocene, Middle Miocene, and Pliocene. These dispersal events likely took place through the North Atlantic Land Bridge, the De Geer Route, and the Bering Land Bridge, and subsequently diverged through sporadic dispersal and vicariance events. Furthermore, our analysis of host associations revealed that the diversification of *Cronartium* species was correlated with their telial‐hosts, and some species may have experienced host jump events, indicating a complex interplay between host specificity and pathogen–host interaction during *Cronartium* evolution.

## Graphical abstract

We determined that *Cronartium* probably occurred around 91.78 Ma during the Upper Cretaceous. *Cronartium* species may originated in Asia and North America and subsequently had several intercontinental dispersals via the North Atlantic Land Bridge, the De Geer Route, and the Bering Land Bridge. Additionally, we found that the diversification of *Cronartium* species was correlated with their telial‐hosts, and some species may have experienced host jump events during their evolution.

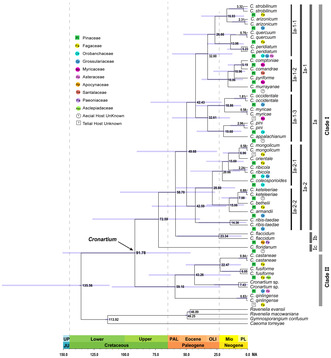

## 1 Introduction

Invasive tree pathogens represent a major threat to natural forests and plantations worldwide and are responsible for their increasing biological destruction (Ghelardini et al. 2017; Santini et al. 2013). Understanding the biogeographical pattern, population structure, and origin of invasive pathogens is crucial for phytosanitary and disease control. Pine stem rusts caused by *Cronartium* (Pucciniales, Cronartiaceae) are the most damaging pine tree disease, particularly the white pine blister rust (WPBR) caused by *Cronartium ribicola* J.C. Fisch, which can affect all white pines (*Pinus* subsection *Strobus*). In severe cases, WPBR can lead to growth reduction and mass mortality because of the difficulty in controlling this disease in large natural stands, resulting in significant economic and ecological losses in pine forests (Brar et al. 2015; McDonald and Hoff 2001; Zhang et al. 2010; Zhao et al. 2022). Due to their tremendous destructive potentials, many *Cronartium* species, including *C. ribicola* , have been listed as quarantine pests by the United States, the European Union, and Asian countries (EPPO 2023).

The taxonomy of *Cronartium* species is challenging due to their overlapping morphology and obscure host range. Up to date, 68 *Cronartium* species have been listed in Index Fungorum (www.indexfungorum.org) (accessed on 18 January 2024); however, some names are invalid and not available in GenBank (Aime et al. 2018). Zhao et al. (2022) identified twenty‐six *Cronartium* species via multi‐gene phylogenetic analysis, morphological characteristics, host specificity, and geographic origins. The same study highlighted the usefulness of aecio‐ and teliospore ornamentation, as well as the aecial peridium, for species delimitation.

White pine blister rust has affected Korean white pine ( *Pinus koraiensis* ) in northeastern China and Chinese white pine ( *Pinus armandii* ) in the Himalayas and central China (McDonald and Hoff 2001; Richardson et al. 2009; Zhang et al. 2010). The blister rust pathogen on *P. armandii* was typically identified as *C. ribicola* or *C. quercuum* based on morphology or telial hosts (Zhuang 2005). However, Li (2008) showed that *Cronartium* species on *P. armandii* (southwestern China) were distantly related to *C. ribicola* on *P. koraiensis* (northeastern China) based on the phylogenetic analysis of the Chinese *Cronartium* populations (Richardson et al. 2009). More recently, Zhao et al. (2022) described a new species, *Cronartium armandii*, on *P. armandii*, of which was phylogenetically and morphologically distant from the two known species. Although the major *Cronartium* species and their phylogenetic relationships with other species in China have been largely elucidated (Zhang et al. 2010; Zhao et al. 2022), the understanding of species variability, as well as the ecological factors and evolutionary processes shaping the distribution patterns in *Cronartium*, are inadequent.

Rust fungi (Pucciniales, Basidiomycota), including *Cronartium*, are obligate biotrophs with a complex life cycle that develop a very close relationship with their aecial‐telial hosts. However, the biogeography and evolutionary history of rust fungi remain poorly understood, which could be hampered by the lack of a reliable fossil record, complex taxonomic relationships, and incomplete sampling in some regions (Li et al. 2020; Schmit and Mueller 2007; Taylor and Berbee 2006). McTaggart et al. (2016) indicated that many rust fungi shared a most recent common ancestor (MRCA), with a mean age of 113–115 Ma, which was much younger than previous estimates of 150–300 Ma (Aime 2006; Leppik 1953). Their results also showed that families, genera, and species of rust fungi within the two suborders Uredinineae sensu Aime (2006) and Melampsorineae *sensu* Aime (2006) diverged approximately 38–46 Ma, 22–37 Ma, and 0.3–17 Ma, respectively, which was not consistent with a study on the time tree of life. In contrast, Aime et al. (2018) estimated the timing of diversification in Pucciniales using multi‐loci sequence data and showed that Melampsorineae has a median age estimate for the most recent common ancestor (tMRCA) of 85 Ma, but in the Melampsorineae datasets. This discrepancy may stem from the fact that representatives of Pucciniastraceae are only included in the Melampsorineae datasets, not from the Pucciniales datasets (only one *Cronartium* species is included in the Pucciniales datasets). In general, among biogeographic studies with divergence times estimation at the rust order and family levels (Aime et al. 2018; McTaggart et al. 2016) as well as assessing co‐diversification between rust fungi and their hosts, only a few studies have focused on the origins and dispersal events of global *Cronartium* species.

Obligate rust fungi were presumed to have co‐evolved with their host plants (Helfer 2014; Savile 1971). However, molecular data has indicated that rust fungi underwent speciation through either divergence alongside their hosts or host jumps. Many rust taxa have formed well‐supported clades corresponding to host species. For example, in Phragmidiaceae, Raveneliaceae, Endoraecium, and Uromycladium (Aime 2006; Aime et al. 2017; McTaggart et al. 2015). At larger scales, host jump, rather than coevolution, was the main driver for the diversification of rust fungi over a long‐term evolutionary period (McTaggart et al. 2016). *Cronartium* species are widely distributed over large parts of Europe, Asia, and North America (McDonald and Hoff 2001; Zhang et al. 2010). In particular, some species of *Cronartium* have transcontinental distribution patterns due to human‐mediated activities, for example, *C. ribicola* (Richardson et al. 2009). The host associations revealed that *Cronartium* species have wide telial‐host ranges, indicating their ability to infect a wide variety of host plants across different families. Knowledge about the evolutionary relationships of other *Cronartium* species and their aecial‐telial hosts is scarce. There remain multiple evolutionary questions regarding the delimitation and diversification of *Cronartium* species.

Given the species diversity and diversification of *Cronartium* species with an extensive distribution range, it is important to understand this genus by combining phylogenetic analysis, biogeographic analysis, and divergence time estimation. The main objectives of this study are: (1) to investigate the species diversity and phylogenetic relationship of *Cronartium* species in China via the nuclear ribosomal genes (ITS and LSU rRNA regions), as part of an ongoing biodiversity study of tree pathogens in China; (2) to determine the divergence time within the *Cronartium* group, and to reconstruct their evolutionary pattern and biogeographic history.

## 2 Materials and Methods

### 2.1 Sample Collection and Morphological Analyses

A total of 180 specimens of pine stem rust from Shaanxi, Shanxi, and other regions of China have been collected between 2019 and 2023. The specimens have been deposited in the Mycological Herbarium of the Museum of Beijing Forestry University in Beijing, China (BJFC). Dried specimens borrowed from Herbarium Mycologicum Academiae Sinicae, Beijing (HMAS), were also included in this study. Host plants, locality, and time of collection are listed in Table S1.

Approximately 50–60 spores of each specimen were mounted on a microscope slide in a drop of lactophenol or lactophenol cotton blue solution. The size and shape of randomly selected spores were determined using an Olympus SZX2‐FOF light microscope (Tokyo, Japan). Telia, aecio‐, and urediniospores were attached to aluminum stubs coated with double‐sided adhesive tape and then gold‐plated using the SCD‐005 sputter coater (Hitachi, Tokyo, Japan) to prepare specimens for an examination of their surface structure. Observations of surface structure were conducted using a Hitachi SU8010 scanning electron microscope (Tokyo, Japan) operated at 3.0 kV (You, Yang, and Tian 2019). Morphological characteristics were compared to the type specimens, original descriptions, and other published descriptions of the involved species (Zhao et al. 2022).

### 2.2 DNA Extraction, Polymerase Chain Reaction, and Sequencing

Genomic DNA was extracted from fresh specimens of rust on different hosts using a QIAamp DNA Microbiome Kit (Qiagen, Hilden, Germany) according to the manufacturer's instructions. Two nuclear ribosomal RNA gene regions, the ITS regions and intervening 5.8S rRNA gene, and the large subunit (LSU) rDNA were amplified, and a nested polymerase chain reaction (PCR) method was employed to improve the amplification. The PCR amplification and sequencing methods and conditions, along with the primer sequences, were described by You, Yang, and Tian (2019) and Zhao et al. (2022). The PCR products were purified and cloned for sequencing (Tsingke, Beijing, China). Sequence data obtained in this study were included and uploaded to GenBank (Table S2).

### 2.3 Sequence Processing and Taxon Selection

The consensus DNA sequences were assembled using SeqMan (http://www.dnastar.com/t‐seqmanpro.aspx), and raw sequences were aligned using MAFFT v.6.0.0 (https://mafft.cbrc.jp/alignment/server/), removing sequences with large differences and untidy segments at both ends.

Also, representative sequences of four major loci from 26 *Cronartium* species were retrieved from the NCBI database: internal transcribed spacer regions (ITS) with 5.8S nrRNA gene, the large subunit (LSU), the small subunit (SSU) rDNA, as well as the cytochrome c oxidase subunit 3 (CO3). These representative sequences aimed to encompass all known major lineages of *Cronartium* as identified across previous studies and were used for subsequent analyses (Moricca et al. 1996; Schoch et al. 2012; Vogler and Bruns 1998; Zhao et al. 2022).

Two multigene datasets were created to explore the phylogenetic relationship, divergence times, and evolutionary history of *Cronartium* species. Dataset I (ITS + LSU) included 61 taxa: 59 samples being ingroup taxa and two sequences from *Revenelia* as outgroups (Table S2); dataset II (SSU‐ITS‐LSU‐CO3) was generated to determine the tMRCA and to reconstruct the evolutionary and biogeographic history of *Cronartium*, which contained 42 *Cronartium* samples from eight countries as ingroups (China, Japan, the United States, Canada, Greece, Russia, Honduras, and Finland), and four fossils records from other families were selected as the reliable calibration point (*Caeoma torreyae*, *Ravenelia evansii*, *Ravenelia macowaniana*, and *Gymnosporangium confusum*) (Table 1).

**TABLE 1:** Species and their sequences of *Cronartium* used for molecular clock and RASP analyses.

Species	Specimen no.	Country	Region^a^	GenBank accession no.			
				SSU	ITS	LSU	CO3
*Cronartium appalachianum*	Ca‐1	USA	B	—	L76484	—	—
*C. arizonicum*	MICH253346	USA	B	OM745897	MK193824	MK208284	OM721322
	MICH301231	USA	B	OM745898	OM746508	OM746340	OM721323
*C. armandii*	HMAS64281	China	A	OM745905	OM746513	OM746345	OM721330
*C. bethelii*	HMAS82418	China	A	OM745915	OM746522	OM746354	OM721340
*C. castaneae*	HMAS18841	China	A	OM745917	OM746524	OM746356	OM721342
	HMAS8970	USA	B	OM745918	OM746525	OM746357	OM721343
*C. coleosporioides*	Ccol‐yh3‐FP	Canada	B	—	JN943207	—	—
*C. comandrae*	MICH253364	USA	B	OM745927	MK193825	MK208293	OM721352
*C. comptoniae*	UBC‐F5871	USA	B	OM745941	OM746545	OM746377	OM721366
*C. flaccidum*	FLAS‐F‐55559	Finland	C	OM745947	OM746551	OM746383	OM721372
	HMAS82720	Russia	C	OM745957	OM746561	OM746393	OM721382
*C. floridanum*	MICH299992	USA	B	OM745976	OM746576	OM746408	OM721401
*C. fusiforme*	HMAS56356	China	A	OM745983	OM746583	OM746415	OM721408
	HMAS9043	USA	B	OM745986	OM746586	OM746418	OM721411
*C. keteleeriae*	HMAS11129	China	A	OM745989	—	OM746421	OM721414
	HMAS638	China	A	OM745990	—	OM746422	OM721415
*C. mongolicum*	HMAS242639	China	A	OM745991	OM746589	OM746423	—
	ZP‐R7	China	A	OM745992	OM746590	OM746424	—
*C. murrayanae*	MICH301494	USA	B	OM745993	OM746591	OM746425	OM721416
*C. myricae*	MICH253485	Canada	B	OM745995	OM746593	OM746427	OM721418
	MICH253505	Canada	B	OM745996	OM746594	OM746428	OM721419
*C. occidentale*	MICH253479	USA	B	OM745997	OM746595	OM746429	OM721420
	MICH253481	USA	B	OM745999	OM746597	OM746431	OM721422
*C. orientale*	HMAS77667	China	A	OM746005	OM746602	OM746436	OM721428
*C. peridiatum*	NYBG267057	USA	B	OM746018	OM746612	OM746446	OM721441
	TSH‐R14230	Japan	A	OM746019	OM746613	OM746447	OM721442
*C. pini*	MD1	Finland	C	—	X83890	—	—
	GREEK1	Greece	C	—	X83908	—	—
*C. pyriforme*	MICH253420	USA	B	OM746023	OM746617	OM746451	OM721446
*C. qinlingense*	HMAS56423	China	A	OM746025	OM746619	OM746453	OM721448
	HMAS74356	China	A	OM746026	OM746620	OM746454	OM721449
*C. quercuum*	MICH253529	Canada	B	OM746027	OM746621	OM746455	OM721450
	MICH253530	Honduras	B	OM746028	OM746622	OM746456	OM721451
*C. ribicola*	ZP‐R524	China	A	OM746037	OM746631	OM746465	OM721460
	UBC‐F5890	Canada	B	OM746050	OM746644	OM746478	OM721473
*C. ribis‐taedae*	HMAS52871	China	A	OM746068	OM746662	OM746496	OM721491
*C. strobilinum*	FLAS‐F‐53222	USA	B	OM746071	MK193823	MK208285	OM721494
	CSt‐2	USA	B	—	L76482	—	—
*Cronartium* sp.	HMAS49226	USA	B	OM746073	OM746666	OM746500	—
	HMAS41544	China	A	OM746074	OM746667	OM746501	OM721495
*Ravenelia macowaniana*	WM3485	South Africa	—	—	KP687429	KP661594	—
*Ravenelia evansii*	PREM61005	South Africa	—	—	KP687425	MG945999	—
*Caeoma torreyae*	DV29.1	USA	—	AY123284	—	AF522183	—
*Gymnosporangium confusum*	20140330‐2	—	—	—	KP261046	KP261047	—

^a^ The geographic regions used for RASP analysis. (A) Asia, (B) North America, (C) Europe, (D) South America.

### 2.4 Phylogenetic Analysis

To reconstruct the phylogenetic relationships of *Cronartium*, we retrieved published sequences together with new collections obtained in this study, and dataset included 61 sequences from two genes: ITS and 28S (Dataset I). Phylogenetic analyses were performed using maximum parsimony (MP), Bayesian inference (BI), and maximum likelihood (ML) analysis, respectively.

Maximum parsimony analysis was performed in PAUP v.4.0b10 (Swofford 2002). Robustness of parsimonious trees was assessed by 1000 bootstrap replications. Additionally, tree length (TL), concordance index (CI), retention index (RI), and rescaled concordance (RC) were calculated. Bayesian analysis was performed using MrBayes 3.2 (Ronquist and Huelsenbeck 2003) with the Markov Chain Monte Carlo (MCMC), and the best‐fit substitution models were estimated using jModelTest v.2.1.7 (Darriba et al. 2012) based on the implementation of the Akaike information criterion. GTR + I + G was selected as the best evolutionary model, and the Markov chains were run for 1,000,000 generations. Maximum likelihood analysis was performed with 1000 bootstrap replicates using RAxML v.8 (Stamatakis 2014) and IQ‐TREE v.1.6.5 (Nguyen et al. 2015), with GTR‐GAMMA as the evolutionary model for site substitution. All trees were visualized using FigTree v.1.4.4 (http://tree.bio.ed.ac.uk/software/figtree/). Furthermore, Kimura's two‐parameter model (K2P) of base substitution implemented in PAUP 4.0b10 (Swofford 2002) was used to calculate intra‐ and interspecific genetic distances.

### 2.5 Molecular Dating Analysis

A major challenge for molecular dating of fungi (Pucciniales) is the lack of fossil records that can be used to calibrate the divergence times for the extant lineage. Consequently, secondary calibrations have been widely adopted to estimate the divergence times of fungi (Floudas et al. 2012; Renner 2005). Calibration of nodes in *Cronartium* in dataset II was used by the following four fossil records: (i) the most ancestral member of the Pucciniales, *Caeoma torreyae*, which occurs aecia on *Torreya californica* diverged 66–250 Ma (Aime 2006; Peterson 1974); (ii) the genus *Ravenelia* (*Ravenelia evansii* & *Ravenelia macowaniana*), which diverged from other members of the rust at the minimum ages of 40.4–55.8 Ma (Ramanujan and Ramachar 1963); (iii) *Gymnosporangium confusum*, the early divergent species of *Gymnosporangium*, were calibrated between 44.3–51.7 Ma (Zhao et al. 2016).

Molecular dating analyses were performed using BEAST v.1.8.4 (Drummond and Rambaut 2007) with XML files created and uploaded to BEAUTi v.1.7.5. Site models for nucleotide sequences were analyzed using the GTR + Gamma substitution. An uncorrelated relaxed clock and lognormal rate distribution were used as the clock model. Tree priors were set to “Yule Speciation.”

Four independent BEAST analyses were performed for 10 million generations respectively, with the posterior distribution of optimal trees sampled every 1000 generations for each run. Tracer v.1.7 (Rambaut et al. 2018) was used to determine the behavior of the chains and to enhance the effective sampling size (ESS) for parameter convergence. The mean node ages and 95% highest posterior density (HPD) values were summarized and annotated on the maximum clade credibility tree using TreeAnnotator v.1.8.4 (Drummond and Rambaut 2007) with a burn‐in of 20%. The Maximum likelihood and Bayesian inference trees were used as the comparison with the MCC tree from BEAST. FigTree v.1.4.4 was used to visualize the resulting tree and to obtain the mean values and the 95% HPD.

### 2.6 Ancestral Distribution Region Reconstruction

Based on the MCC tree from BEAST analysis of Dataset II, we employed a rigorous ancestral area reconstruction analysis using the statistical Bayesian Binary Markov chain Monte Carlo model (BBM) and dispersal‐vicariance analysis (S‐DIVA) in RASP v.4.X (Yu, Blair, and He 2020) to elucidate the biogeography of *Cronartium*. The geographical distribution areas of *Cronartium* were defined as four distinct regions: (A) Asia, (B) North America, (C) Europe, and (D) South America. Each taxon in our dataset was allocated to a geographic region based on its presently recognized distribution range.

To ensure accurate results in the BBM analysis, the MCMC chain was run for 10 million generations with a sampling frequency of 100. For the S‐DIVA analysis, we employed the “Allow Reconstruction” option allowing up to 100 reconstructions and two random steps initially, followed by performing up to 1000 reconstructions on the final tree. Each node was permitted a maximum of four regions. The optimal S‐DIVA reconstruction is summarized based on the pruned maximum clade credibility tree derived from our comprehensive phylogenetic analysis.

## 3 Results

### 3.1 Phylogenetic Relationships

**FIGURE 1:**
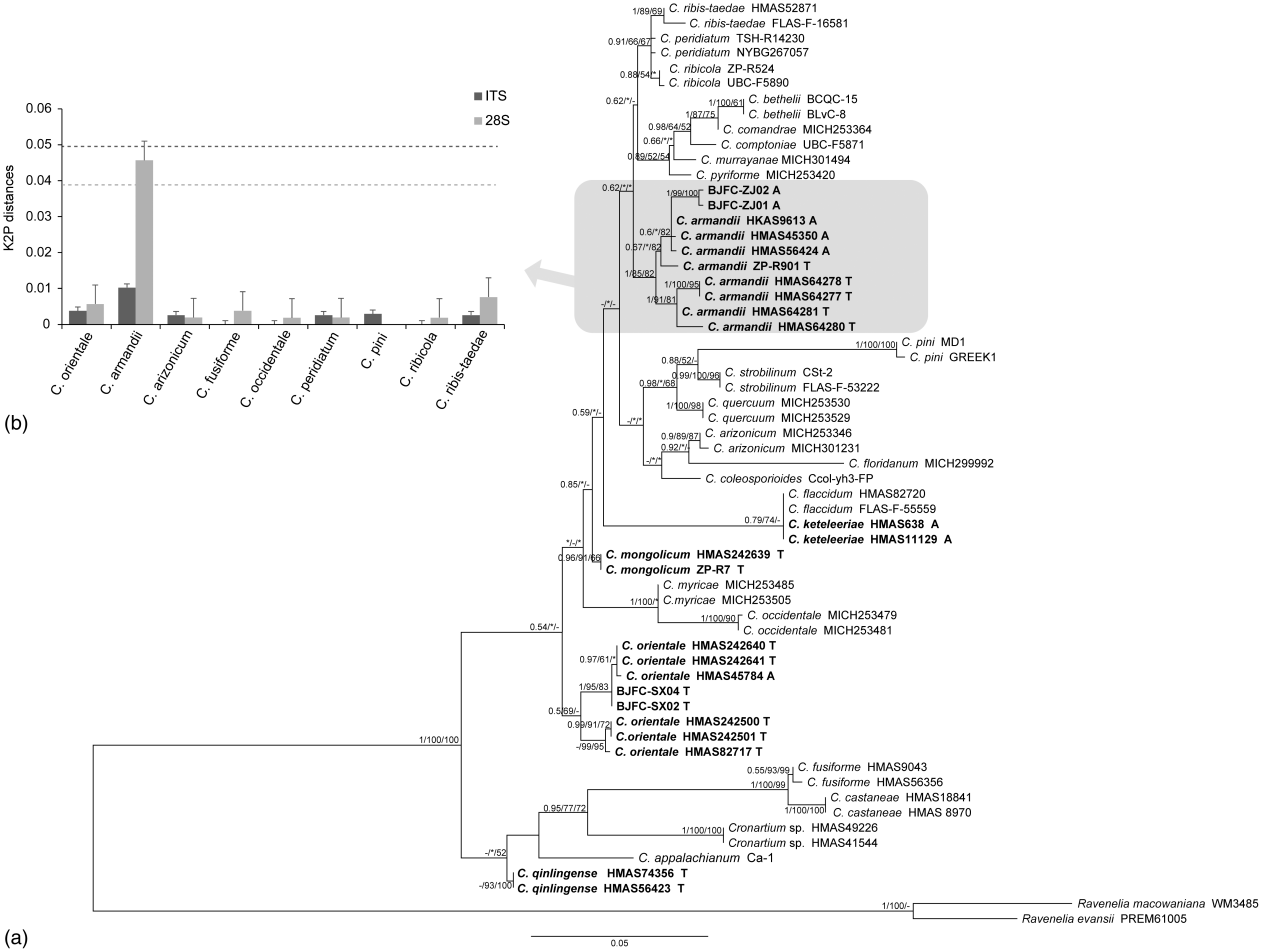
(a) Phylogenetic relationship of the *Cronartium* species based on ITS+ LSU rDNA dataset. (dataset I). One of the best trees was shown with statistical support indicated at nodes. Bootstrap values were calculated from 1000 replications. ML, BI, and MP ≤ 50% are indicated by ‘*’, and ‘‐’ means that the support rate of the node is not given in the BI or ML topology. The scale bar represents the number of base substitutions per site. *Cronartium* species that only occurred in China are in bold. (b) Intraspecific distances obtained within each *Cronartium* species (genetic distance of 0 is not shown); the horizontal line corresponds to the mean intraspecific distance obtained over the entire dataset.

The phylogenetic tree of all representative *Cronartium* species, based on the concatenated ITS and LSU rRNA sequence datasets (dataset I), comprised 59 ingroups and two outgroups, *Ravenelia* sp. (WM3485 and PREM61005). A total of 1114 characters including gaps (489 for ITS rRNA and 608 for LSU rRNA), with 748 constant characters and 99 parsimony‐uninformative variable characters, were included in the phylogenetic analysis. Similar tree topologies were obtained by MP, ML, and BI methods. The phylogram based on the ML analysis was plotted in Figure 1.

The phylogenetic tree indicated that our collections can be divided into two well‐supported monophyletic clades that include two known species, *C. armandii* and *C. orientale*. The rust fungi on *P. armandii* collected from Shanxi Province, China, clustered with the holotype (HMAS45350) of *C. armandii* with high support values (1/85/82), were therefore described as *C. armandii* . All the specimens collected on *Quercus* sp. from Shaanxi Province clustered together and nested within a clade that contained *C. orientale* , and they were considered to be the same species. All the samples collected on *Quercus mongolica* from Heilongjiang province were identified as *C. mongolicum* based on morphology and phylogeny.

Deep intraspecific variation was observed in *C. armandii* lineage, showing a larger intraspecific variation than the average for 28S rRNA (K2P = 0.0457 ± 0.0157). 28S rRNA readily differentiated five strains of *C. armandii* found on aecial host *P. armandii* , and five other *C. armandii* strains occurring on telial host *Ribes* sp. in a well‐supported group.

### 3.2 Molecular Dating Analysis

**FIGURE 2:**
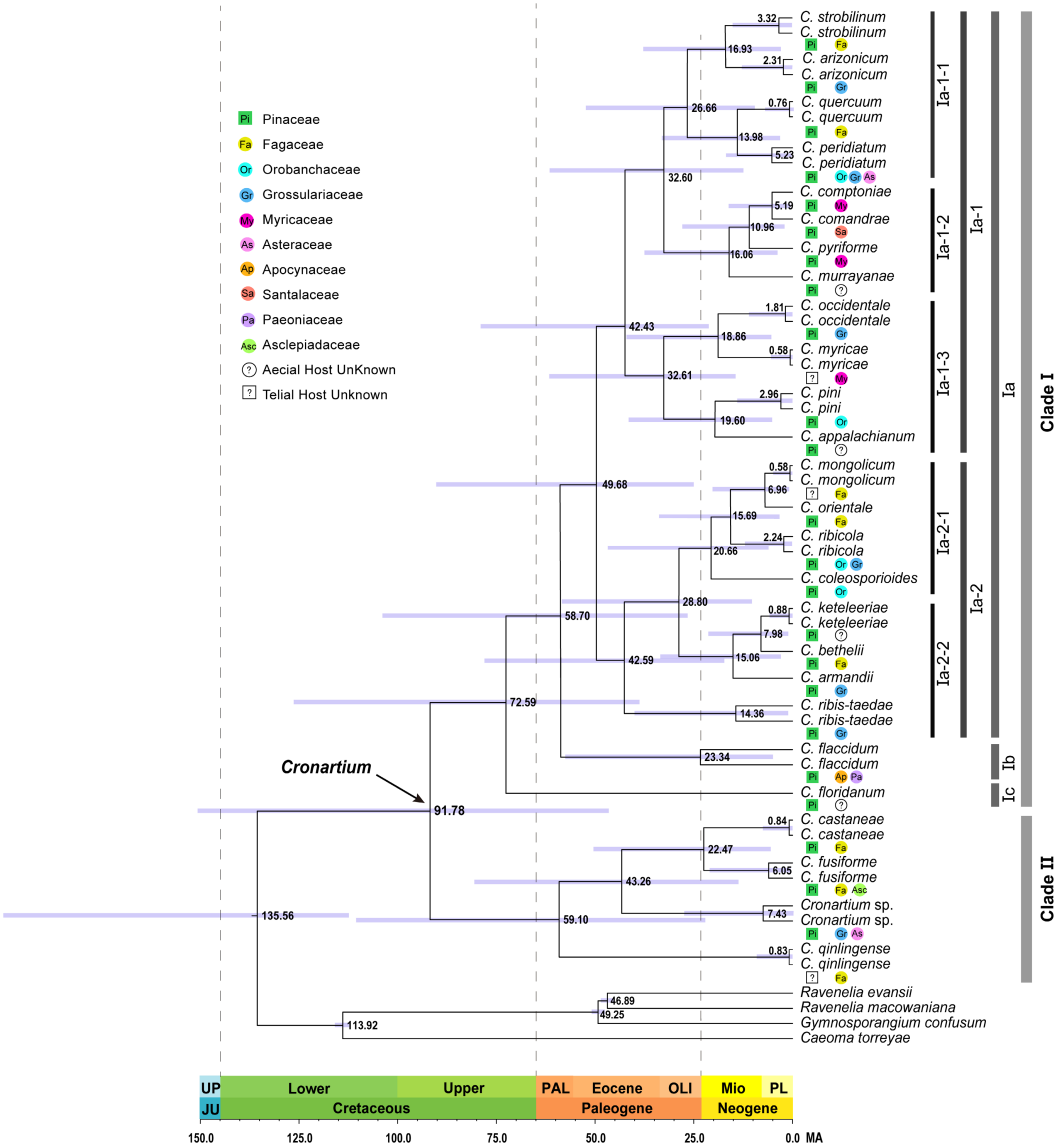
Divergence time estimates by BEAST analyses for major nodes of *Cronartium* species. The value at the node represents the divergence time (unit: Ma). 95% HPD is represented by blue‐purple bands on the branches.

The Maximum Clade Credibility (MCC) tree generated by BEAST analysis was identical to the Maximum Likelihood (ML) and Bayesian inference (BI) phylogeny based on the concatenated sequences of SSU‐ITS‐LSU‐CO3 (dataset II) (Figures S1 and S2). Molecular dating using four genes estimated that the tMRCA of the genus *Cronartium* was at 91.78 Ma (95% HPD: 46.80–150.90 Ma) during the Upper Cretaceous (Figure 2). The diversification of Clade I was estimated to have occurred around 72.59 Ma (95% HPD: 38.76–126.31 Ma), while Clade II diversified around 59.10 Ma (95% HPD: 22.47–110.88 Ma). The earliest diverging branch within Clade I was represented by *C. floridanum* (Clade Ic), which originated from North America, with an estimated divergence time of 72.59 Ma in the late Cretaceous, followed by the split of the Clade Ib and Clade Ia at 58.70 Ma (95% HPD: 26.57–103.85 Ma) during the Upper Cretaceous. The root node of the species‐rich Clade Ia, comprising over two‐thirds of *Cronartium* species (20/26), was dated at 49.68 Ma (95% HPD: 24.62–89.83 Ma) in the middle Eocene. *C. qinlingense* in Clade II separated from its tMRCA during the late Paleocene (59.10 Ma, 95% HPD: 22.47–110.88 Ma). Three lineages of Clade II diverged during the middle Eocene (43.26 Ma, 95% HPD: 13.69–80.58 Ma). Our results also indicated that the diversification of most *Cronartium* species occurred during the Miocene to Pliocene epochs.

The telial hosts for *Cronartium* taxa were deduced from all available literature (McDonald and Hoff 2001; Richardson et al. 2009; Zhang et al. 2010; Zhao et al. 2022) and were assigned at the family level, particularly in cases where a *Cronartium* species was known to associate with multiple genera within a host family or two host families. The results of the host associations revealed that *Cronartium* species had wide telial‐host ranges, indicating their ability to infect a wide variety of host plants across different families. The ancestral lineage, *C. qinlingense*, found on *Quercus aliena* in China, was identified as closely related to three species within Clade II: *C. castaneae* , *C. fusiforme*, and *Cronartium* sp., which are distributed across Asia and North America. Among these, *C. qinlingense*, *C. castaneae* , and *C. fusiforme* all occurred on species of *Quercus* sp. or *Castanea* sp. in Fagaceae. However, unlike the mentioned species previously, *Cronartium* sp. was observed on *Ribes* sp. and *Saussurea* sp., members of the Grossulariaceae and Asteraceae family respectively.

*C. floridanum* on *Pinus palustris* from the United States diversified from the most ancestral member of the *Cronartium* at 72.59 Ma, but the telial hosts of *C. floridanum* remain unknown. In Clade Ia‐1‐2, host species within the Myricaceae family were recorded as telial hosts for *C. comptoniae* and *C. pyriforme* . However, the closely related species *C. comandrae* was found to occur on hosts within the Santalaceae family. As for *C. murrayanae*, the telial host remains unknown. Within Clade Ia‐2‐1, two closely related species, *C. mongolicum* and *C. orientale* , were found exclusively on hosts within the Fagaceae family. The ancestral species in Clade Ia‐2‐1, *C. coleosporioide*s, was identified on hosts within the Orobanchaceae family. Notably, *C. ribicola* primarily occurred on hosts within the Orobanchaceae and Grossulariaceae families.

### 3.3 Ancestral Distribution Region Reconstruction

**FIGURE 3:**
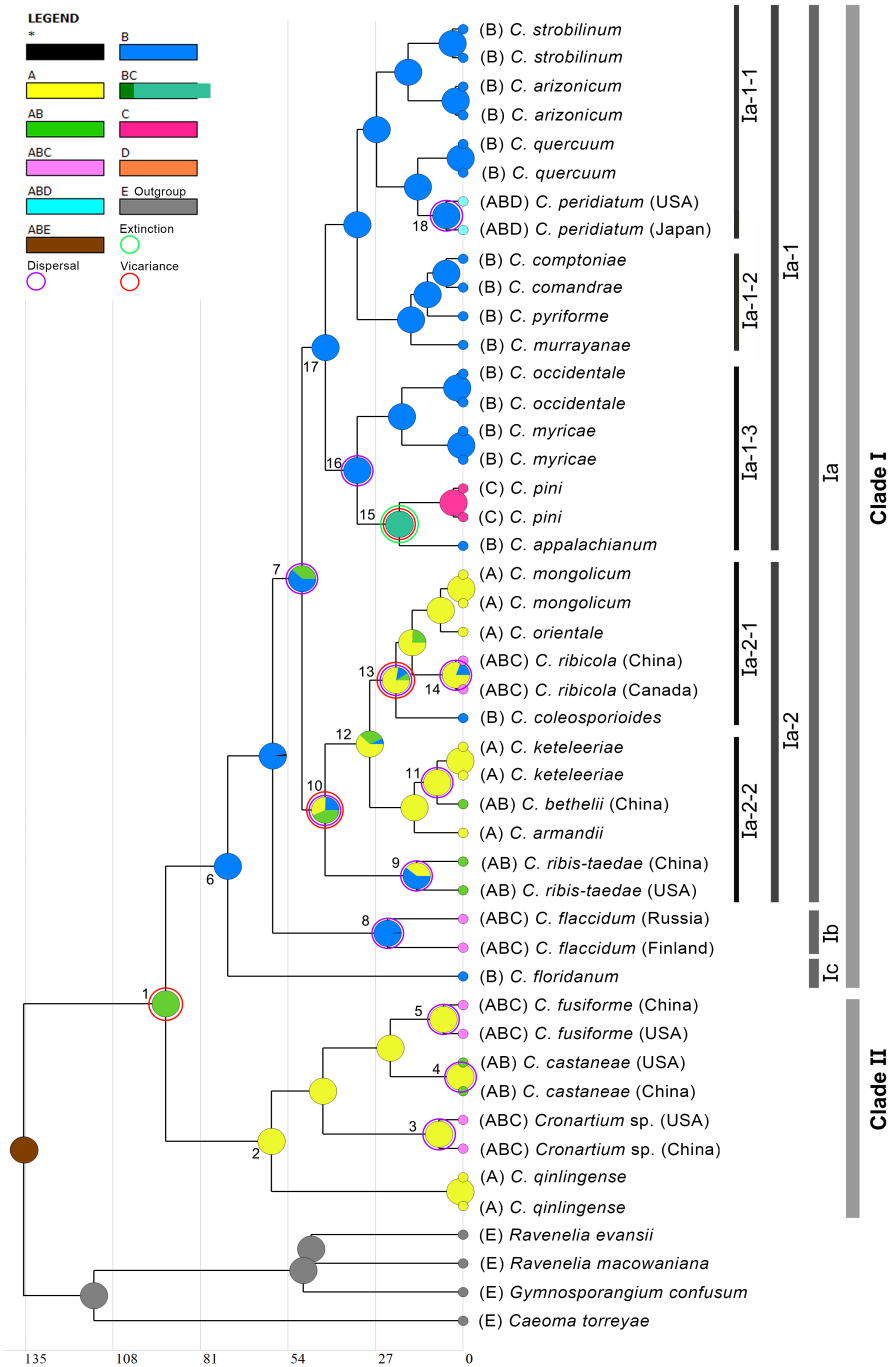
Ancestral area estimation of *Cronartium* using the S‐DIVA model by RASP. Geographic distribution areas: (A) Asia, (B) North America, (C) Europe, (D) South America. The proportion of colors in a node circle represents the probability of each area to be a historical distribution region.

The optimized ancestral distribution regions inferred for internal nodes of *Cronartium* (dataset II) are not fully consistent in the BBM and S‐DIVA analyses (Table 2). In the S‐DIVA analyses (Figure 3), it was revealed that Asia and North America were the regions of origin for *Cronartium*. Clades II was estimated to originate from Asia, which implied that several dispersal events in Europe and North America took place during the late Paleocene and early Miocene. North America was estimated to be the most probable ancestral area for species in Clade I. The basal lineage *C. floridanum* (node 6) from North America appeared isolated from ancestral *Cronartium* species evolving in the remaining regions. Clade Ia‐1 and Clade Ia‐2 were estimated to have originated from North America and North America & Asia, respectively. Most species in Clade Ia‐1 appeared to be restricted to a single area, North America. However, one species (*C. peridiatum*) occupied three areas (North America, Asia & South America), and another species ( *C. pini* ) was present only in Europe. In Clade Ia‐2, several dispersal events to Asia occurred during the middle Eocene and Neogene (Figure 3, Table 2). In the BBM analysis, Asia was indicated to be the region of origin for *Cronartium*, although the relative probability for this node was very low (Table 2). The ancestral areas for most nodes in BBM were estimated as multiple‐regional origins, covering Asia, North America, and Europe, for example, nodes 3, 5, 8, and 14. However, similar ancestral areas for some nodes were inferred by the BBM and S‐DIVA analyses, such as nodes 2, 10, 11, 16, and 17.

**TABLE 2:** Divergence time for major nodes of *Cronartium*, with results of ancestral area estimation using the BBM and S‐DIVA models.

Clade	Node	Age estimation (Ma)			Ancestral area reconstruction			
		Median age	95% HPD	Posterior probability	BBM^a^		S‐DIVA^b^	
					Area	Probability	Area	Probability
—	1	91.78	46.80–150.90	1	A	0.37	AB	1.0
Clade II	2	59.10	22.47–110.88	1	A	0.45	A	1.0
	3	7.43	0.23–27.88	1	ABC	0.98	A	1.0
	4	0.84	0.00–7.49	1	AB	0.97	A	1.0
	5	6.05	0.47–21.00	1	ABC	0.93	A	1.0
Clade I	6	72.59	38.76–126.31	1	AB	0.52	B	1.0
	7	49.68	24.62–89.83	0.99	AB	0.84	B	0.62
	8	23.34	4.96–57.55	1	ABC	0.89	B	0.99
	9	14.36	1.12–40.00	1	AB	1.0	B	0.61
	10	42.59	17.30–78.02	1	AB	0.97	AB	0.44
	11	7.98	1.09–21.30	0.99	A	0.6	A	1.0
	12	28.80	10.21–58.33	0.89	AB	0.85	A	0.62
	13	20.66	5.75–46.45	0.52	AB	0.75	A	0.78
	14	2.24	0.03–11.94	1	ABC	0.89	A	0.81
	15	19.60	5.16–41.51	0.97	B	0.97	BC	0.13
	16	32.61	14.42–61.60	1	B	0.98	B	1.0
	17	42.43	21.21–78.98	0.99	B	0.88	B	1.0
	18	5.23	0.19–16.84	1	ABD	0.83	B	1.0

^a^ Bayesian Binary Markov Chain Monte Carlo model.

^b^ Statistical Analysis of Diffusion Isolation model.

## 4 Discussion

In our study, 26 different *Cronartium* species are divided into two distinct clades based on phylogenetic analysis of ITS and 28S loci. The tree topologies based on these two ribosomal genes were similar to the data derived from the combined ribosomal gene and CO3 loci (SSU‐ITS‐LSU‐CO3) (Figures S1 and S2), suggesting that the nuclear ribosomal loci provided comparable resolution compared to the combined loci, which also included the mitochondrial gene. Within *C. armandii* , genetically distinct lineages were identified (Figure 1b). It is possible that *C. armandii* has undergone faster or more recent speciation compared to the genetically conserved species. The observed pattern of host associations among *Cronartium* species implied that species diversification within *C. armandii* could be influenced by various factors, including specialization to different *Ribes* species as telial hosts or adaptation to specific pine species such as *P. armandii* as aecial hosts. Conversely, some species (e.g., *C. castaneae* , *C. flaccidum* , *C. mongolicum* , *C. myricae* , *C. qinlingense*, and *C. quercuum*) exhibited no or little genetic variation. Further investigation with larger sample sizes may shed light on whether this pattern persists.

The broad phylogenetic sampling of *Cronartium* taxa and sequence data allowed a thorough estimate of the diversification and timing of evolutionary events. Our data indicated that divergence of the genus *Cronartium* probably occurred in the Upper Cretaceous (91.78 Ma, Figure 2), which is much older than the recent *Cronartium* age estimates proposed by Aime, Bell, and Wilson (2018), who estimated the divergence of *Cronartium* in mid Paleogene (27.73 Ma) based on limited samples—four *Cronartium* species and four related *Pucciniastrum* species. The discrepancy could be attributed to the inclusion of all *Cronartium* taxa in our study, whereas only four *Cronartium* species were included in previous study. Aime, Bell, and Wilson (2018) indicated that two *Cronartium* species, *C. ribicola*, and *C. flaccidum*, splitted from their closest relatives around 18.06 Ma, which is consistent with those from our study. In our analysis, *C. ribicola* diverged from its closest relatives around 15.69 Ma (95% HPD: 5.74–46.54 Ma) (Figure 2). The increased number of taxa, coupled with the restricted availability of sequence data used in age estimations, may lead to younger estimate during the molecular dating (Aime, Bell, and Wilson 2018). The Melampsorineae, comprising the sequences data of five rust species, *Cronartium quercuum = Endocronartium harknessii*, *Coleosporium plumeriae*, *Coleosporium eupatorii*, *Chrysomyxa arctostaphyli*, and *Melampsora medusae*, had a tMRCA median age estimate of 85.54 Ma (95% HPD: 66.40–109.10 Ma) in the Pucciniales analysis. However, the median age of the corresponding clade in the Melampsorineae dataset, comprising three *Cronartium* species (*C. ribicola, C. flaccidum*, and *C. quercuum*), twenty‐three *Coleosporium* species, sixteen *Chrysomyxa* species, and some *Melampsora* and *Pucciniastrum* species, was 57.06 Ma (95% HPD: 26.32–96.65 Ma). Additional genome characterization of the rust fungi is also needed to accurately estimate the diversification times of major lineages (Floudas et al. 2012).

The tMRCA to all extant *Cronartium* diversified around 91.78 Ma (HPD: 46.80–150.90 Ma), which occurred after the Pinaceae, estimated to have arisen between 170.0 Ma (95% HPD: 101.1–194.7 Ma) (Kumar et al. 2022). The most ancestral member of the *Cronartium* recovered by this study was *C. floridanum* from the United States, which diverged approximately 72.59 Ma (95% HPD: 38.76–126.31 Ma). It exclusively infected *Pinus* as its aecial host, which diverged around 130.2 Ma (95% HPD: 81.0–140.0 Ma) (Kumar et al. 2022), and this rust fungus was simply not present at that time.

*Cronartium* species are host‐specific with extremely complex life cycles, and they produce the spermogonia and aecia on Pinaceae (*Pinus* sp.), while the uredinia and telia on several families of angiosperms, such as Fagaceae, Myricaceae, Asclepiadaceae, and Santalaceae. Aime, Bell, and Wilson (2018) indicated that the emergence of early diversifying lineages of rust fungi corresponds to the ages of coniferous hosts, while the majority of extant rust fungi diversity appears to have co‐evolved with their angiosperm hosts during the Cretaceous period. Usually the aecial stages of *Cronartium* are confined to one host family (Pinaceae), with a wider telial host range across several families of angiosperms. Furthermore, our combined molecular dating analysis showed a correlation between the diversification of the *Cronartium* species and the telial hosts. Species of the angiosperm family Fagaceae diversified around 57.2 Ma (95% HPD: 25.2–77.4 Ma) (Kumar et al. 2022), and its rust, *C. qinglingense*, recovered as the most ancestral member found on Fagaceae, diverged at 59.10 Ma (Figure 2), which correlates with the evolutionary divergence times for Fagaceae. Based on molecular dating and host reconciliation analyses, McTaggart et al. (2016) and Aime, Bell, and Wilson (2018) indicated that host jumps, whether occurring across large taxonomic groups or between genera within the same family, likely played a significant role in shaping the diversity of rust genera. Similarly, *Cronartium* species diversified either through host shifts, facilitated by coevolution, or via subsequent host jumps. In Clade II, *Cronartium* sp. from Asia and North America infects *Ribes* sp. in Grossulariaceae, which is the only example of a species of *Cronartium* on a host outside of the family of Fagaceae. This may represent an example of a host jump. The two rusts in Clade Ia‐2‐1, *C. mongolicum* , and *C. orientale* , have diversified on Fagaceae after its relatively recent split from the ancient North American species *C. coleosporioides* on hosts of the Orobanchaceae family. Leppik (1953) proposed that their plant hosts could have shaped the evolution of rust fungi, and the alternating stages of biological specialization (BSp) and biogenic radiation (BgR) are integral to the evolutionary dynamics of rust fungi. McTaggart et al. (2016) and Aime, Bell, and Wilson et al. (2018) demonstrated that the aecial host is under the strongest selective pressure for conserving host associations (BSp), while the telial host stage experiences less stringent constraints and may play a more significant role in speciation events (BgR). A host reconciliation analysis in the genus *Cronartium* would be necessary to understand and determine if the observed host jumps are indeed occurring during the telial stage and if they are indicative of BgR driving speciation within the *Cronartium* lineage.

Clade Ia, characterized by its rich species diversity, diverged from its closest relatives at approximately 49.68 Ma, during the middle Eocene period (Figure 2). This period closely aligned with the occurrence of the second Eocene cooling, which occurred between 33.9 and 56 Ma. This global cooling event had profound effects on the distribution and evolution of numerous plant species, particularly those within the Pinaceae family. It likely prompted some *Cronartium* species to adapt to colder temperatures, consequently facilitating the expansion of *Cronartium* species throughout the Northern Hemisphere. Furthermore, our findings suggested that both Asia and North America could be the regions of origin for *Cronartium*. The biogeographic pattern observed in the distribution of *Cronartium* taxa between Asia and North America can be explained by the Dispersal‐Vicariance disjunction pattern, a well‐documented phenomenon observed in numerous angiosperm and fungal taxa present in both regions (Li et al. 2020; Yi, Jin, and Wen 2015). The earliest diverging lineage in Clade Ia was inferred to have originated in North America during the middle Eocene (49.68 Ma: Figure 3; Table 2), and the earlier dispersal to Europe and Asia via the North Atlantic Land Bridge, the De Geer route, and the Bering Land Bridge, which were exposed during periods of low sea level (Lickey, Hughes, and Petersen 2002). The diversification and intercontinental dispersals of most *Cronartium* species within Clade Ia‐2 primarily occurred during the middle Miocene to Pliocene epochs. This timeline indicated that a migration event from North America to Asia might have occurred via Beringia, forming an ancient range throughout the Northern Hemisphere. Subsequently, allopatric divergence may have driven along with the intermittent disappearance of Beringia. The intercontinental distribution of certain *Cronartium* species, such as *C. castaneae* and *C. fusiforme*, across Asia, Europe, and North America, could be indicative of dispersal via Beringia during the late Miocene and middle Pliocene. Subsequent vicariance events, such as the opening of the Bering Strait and the disappearance of the North Atlantic Land Bridge, likely acted as barriers to gene flow between populations of the *Cronartium* species within Clade II. These geological changes would have restricted the movement of individuals between continents, leading to increased genetic isolation and promoting the divergence of *Cronartium* populations. Additional studies on the genomes of the representatives in different regions and on aecial‐telial hosts would help to elucidate the mechanism of speciation and adaptation.

## Author Contributions

**Jingyu Zhang:** methodology (lead), writing – original draft (lead). **Clement K. M. Tsui:** writing – review and editing (equal). **Chongjuan You:** writing – review and editing (equal).

## Conflicts of Interest

The authors declare no conflicts of interest.

## Supporting information


**Figure S1.** Maximum likelihood tree for molecular clock analysis based on IQ‐TREE.

**Figure S2.** Bayesian inference tree for molecular clock analysis based on MrBayes.

**Table S1.** *Cronartium* samples collected and used for analysis in this study.

**Table S2.** Species and their sequences of *Cronartium* used for phylogenetic analysis.

## Data Availability

Data from this study are available in the article or in the Supporting Information.
