# Supplementary figures and images for: Species Diversity, Host Association, and Evolutionary History of Cronartium: An Important Global Fungal Pathogen to Trees
Source: Ecol Evol. 2024 Nov 10;14(11):e70545. doi: 10.1002/ece3.70545 (PMC11551067; doi:10.1002/ece3.70545)

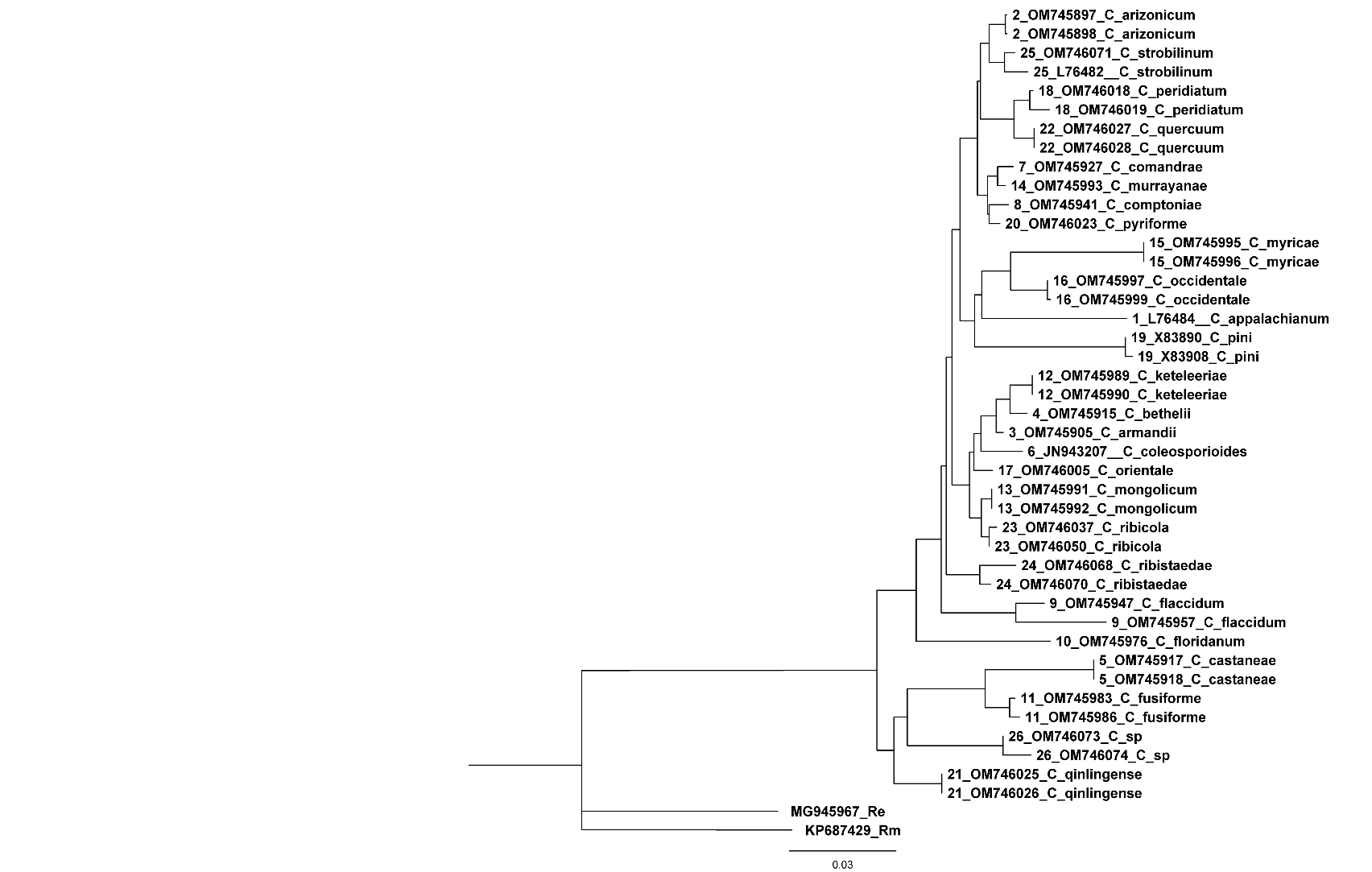


Fig.S1 Maximum likelihood tree for molecular clock analysis based on IQ-TREE.

Supplement: Supplementary file 1 — Figure S1. Maximum likelihood tree for molecular clock analysis based on IQ‐TREE. [file ECE3-14-e70545-s001.docx]

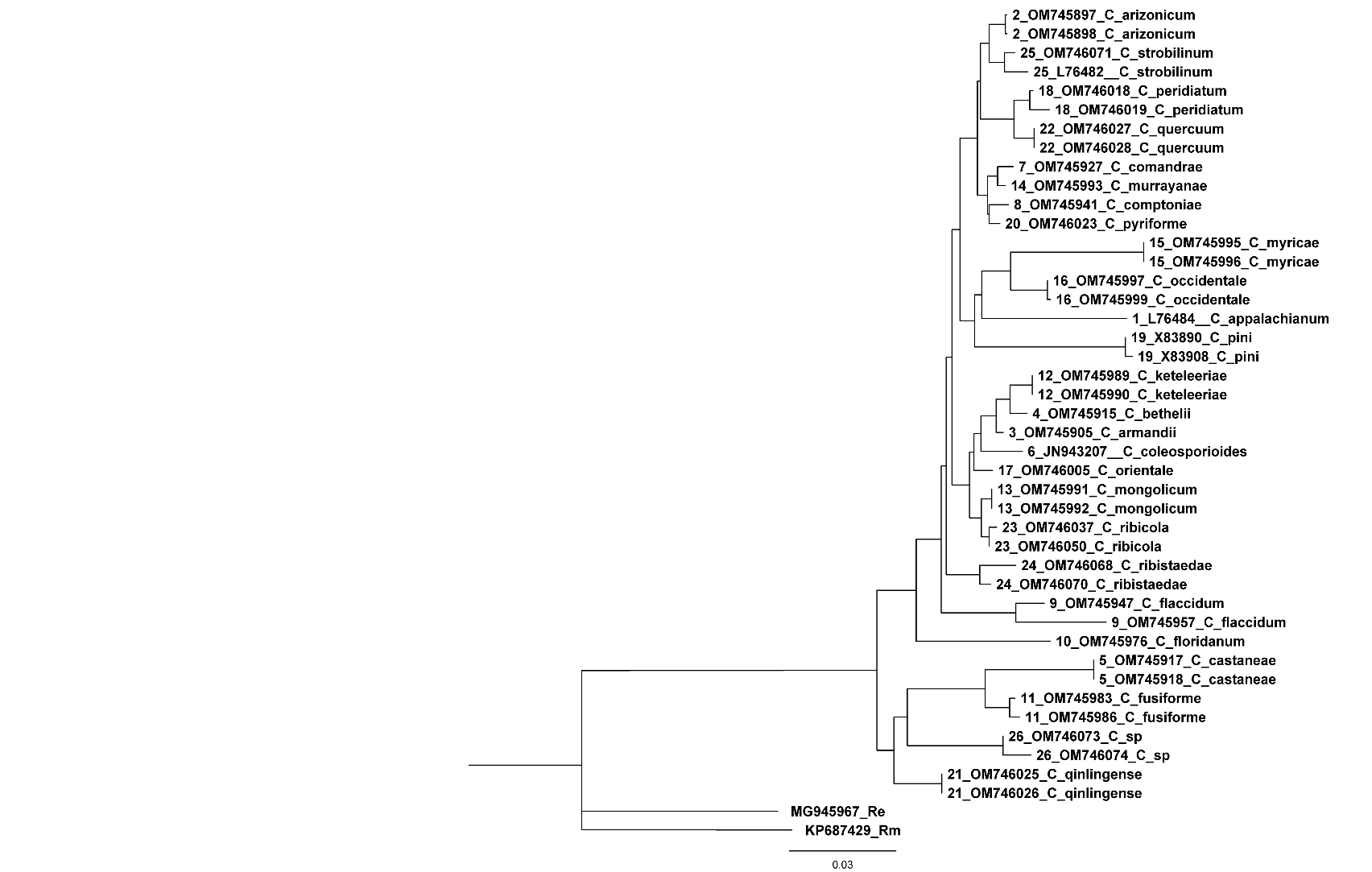


Fig.S2 Bayesian inference tree for molecular clock analysis based on MrBayes.

Supplement: Supplementary file 2 — Figure S2. Bayesian inference tree for molecular clock analysis based on MrBayes. [file ECE3-14-e70545-s003.docx]
