# Supplementary material for: Species Diversity, Host Association, and Evolutionary History of Cronartium: An Important Global Fungal Pathogen to Trees
Source: Ecol Evol. 2024 Nov 10;14(11):e70545. doi: 10.1002/ece3.70545 (PMC11551067; doi:10.1002/ece3.70545)
Supplement: Supplementary file 3 — Table S1. Cronartium samples collected and used for analysis in this study. [file ECE3-14-e70545-s004.docx]

Table S1 *Cronartium* samples collected and used for analysis in this study

| Species | Specimen No. | Host | Location | Date collected | Altitude | Number of collections |
| --- | --- | --- | --- | --- | --- | --- |
| *Cronartium armandii* | HMAS64281 | *Ribes orientale* | Jilong, Tibe, China | 1990.9 | - | 1 |
|  | HMAS64280 | *R. orientale* | Jilong, Tibe, China | 1990.9 | - | 1 |
|  | HMAS64277 | *R. griffithii* | Jilong, Tibe, China | 1990.9 | - | 1 |
|  | HMAS64278 | *R. griffithii* | Jilong, Tibe, China | 1990.9 | - | 1 |
|  | ZP-R901 | *Ribes* sp. | China | - | - | 1 |
|  | HMAS56424 | *Pinus armandii* | Ningshan, Shaanxi, China | 1966.4 | - | 1 |
|  | HMAS45350 | *P. armandii* | Lushi, Henan, China | 1982.5 | - | 1 |
|  | HKAS9613 | *P. corensis* | China | - | - | 1 |
|  | BJFC-ZJ01 | *P. armandii* | Linfen, Shanxi, China | 2022.7 | 1031 | 11 |
|  | BJFC-ZJ02 | *P. armandii* | Yuncheng, Shanxi, China | 2023.7 | 1321 | 8 |
|  | BJFC-ZJ03 | *P. armandii* | Yuncheng, Shanxi, China | 2020.7 | 1321 | 2 |
|  | BJFC-ZJ04 | *P. armandii* | Yuncheng, Shanxi, China | 2021.7 | 1321 | 4 |
|  | BJFC-ZJ05 | *P. armandii* | Yuncheng, Shanxi, China | 2023.7 | 1290 | 1 |
|  | BJFC-ZJ06 | *P. armandii* | Yuncheng, Shanxi, China | 2019.7 | 1137 | 7 |
|  | BJFC-ZJ07 | *P. armandii* | Yuncheng, Shanxi, China | 2022.7 | 1145 | 9 |
| *C. castaneae* | HMAS18841 | *Castanea* sp. | Nanjing, China | 2023.7 | - | 1 |
| *C. fusiforme* | HMAS56356 | *Quercus variabilis* | Shaanxi, China, | - | - | 1 |
| *C. keteleeriae* | HMAS11129 | *Keteleeria davidiana* | Kunming, Yunnan, China | 1943.6 | - | 1 |
|  | HMAS638 | *K. davidiana* | Kunming, Yunnan, China | 1938.6 | - | 1 |
| *C. mongolicum* | HMAS242639 | *Q. mongolica* | Greater Khingan Range, Huma, Hei Longjiang, China | 2000.7 | - | 1 |
|  | ZP-R7 | *Q. mongolica* | Huma, Hei Longjiang, China | 2015.9 | - | 1 |
|  | BJFC-ZJ08 | *Q. mongolica* | Huma, Hei Longjiang, China | 2021.9 | 267 | 19 |
|  | BJFC-ZJ09 | *Q. mongolica* | Huma, Hei Longjiang, China | 2021.9 | 277 | 8 |
|  | BJFC-ZJ10 | *Q. mongolica* | Huma, Hei Longjiang, China | 2020.9 | 203 | 13 |
|  | BJFC-ZJ11 | *Q. mongolica* | Huma, Hei Longjiang, China | 2020.9 | 210 | 8 |
|  | BJFC-ZJ12 | *Q. mongolica* | Huma, Hei Longjiang, China | 2019.9 | 214 | 10 |
|  | BJFC-ZJ13 | *Q. mongolica* | Huma, Hei Longjiang, China | 2022.9 | 203 | 7 |
| *C. orientale* | HMAS242640 | *Q. aquifolioides* | Bomi, Tibet, China | 2010.7 | 3900 | 1 |
|  | HMAS242641 | *Q. aquifolioides* | Bomi, Tibet, China | 2010.7 | 3900 | 1 |
|  | HMAS45784 | *P. densata* | Bomi, Tibet, China | 1983.7 | 736 | 1 |
|  | HMAS242500 | *Q. variabilis* | Hainan, China | 1992.9 | 400 | 1 |
|  | HMAS242501 | *Q. variabilis* | China | - | - | 1 |
|  | HMAS82717 | *Q. glandulifera* | Jiangxi, China | 1996.10 | - | 1 |
|  | BJFC-SX01 | *Quercus* sp. | Ningshan, Shaanxi, China | 2021.7 | 1081 | 9 |
|  | BJFC-SX02 | *Quercus* sp. | Ningshan, Shaanxi, China | 2022.7 | 1071 | 7 |
|  | BJFC-SX03 | *Quercus* sp. | Huoditang, Ningshan, Shaanxi, China | 2023.7 | 1550 | 6 |
|  | BJFC-SX04 | *Quercus* sp. | Huoditang, Ningshan, Shaanxi, China | 2021.7 | 1527 | 6 |
|  | BJFC-SX05 | *Quercus* sp. | Huoditang, Ningshan, Shaanxi, China | 2022.7 | 1525 | 4 |
|  | BJFC-SX06 | *Quercus* sp. | Huoditang, Ningshan, Shaanxi, China | 2019.7 | 1534 | 7 |
|  | BJFC-SX07 | *Quercus* sp. | Huoditang, Ningshan, Shaanxi, China | 2020.7 | 1617 | 9 |
| *C. qinlingense* | HMAS56423 | *Q. aliena* | Qinling Mountains, Ningshan, Shaanxi, China | 1976.8 | - | 1 |
|  | HMAS74356 | *Q. aliena* | Qinling Mountains, Foping, Shaanxi, China | 1991.9 | - | 1 |
| *C. ribicola* | ZP-R524 | *R. nigrum* | Altay Prefecture, Xinjiang, China | 2016.8 | - | 1 |
| *C. ribis-taedae* | HMAS52871 | *R. nigrum* | Xinjiang, China | 1986.8 | - | 1 |
| *Cronartium* sp. | HMAS41544 | *Saussurea bullockii* | Wuyishan, Fujian, China | 1980.9 | - | 1 |
