# Supplementary material for: Species Diversity, Host Association, and Evolutionary History of Cronartium: An Important Global Fungal Pathogen to Trees
Source: Ecol Evol. 2024 Nov 10;14(11):e70545. doi: 10.1002/ece3.70545 (PMC11551067; doi:10.1002/ece3.70545)
Supplement: Supplementary file 4 — Table S2. Species and their sequences of Cronartium used for phylogenetic analysis. [file ECE3-14-e70545-s002.docx]

Table S2 Species and their sequences of *Cronartium* used for phylogenetic analysis.

| **Species** | **Specimen No.** | **Country** | **Spore stage** | **Host** | **GenBank Accession No.** | |
| --- | --- | --- | --- | --- | --- | --- |
|  |  |  |  |  | **ITS** | **LSU** |
| *Cronartium appalachianum* | Ca-1 | USA | 0, I | *Pinus virginiana* | L76484 |  |
| *C. arizonicum* | MICH253346 | USA | II, III | *Castilleja linariaefolia* | MK193824 | MK208284 |
|  | MICH301231 | USA | 0, I | *P. ponderosa* | OM746508 | OM746340 |
| *C. armandii* | HMAS64281 | China | II, III | *R. orientale* | OM746513 | OM746345 |
|  | HMAS64280 | China | II, III | *R. orientale* | OM746514 | OM746346 |
|  | HMAS64277 | China | II, III | *R. griffithii* | OM746516 | OM746348 |
|  | HMAS64278 | China | II, III | *R. griffithii* | OM746515 | OM746347 |
|  | ZP-R901 | China | II, III | *Ribes* sp. | OM746518 | OM746350 |
|  | HMAS56424 | China | 0, I | *P. armandii* | OM746519 | OM746351 |
|  | HMAS45350 | China | 0, I | *P. armandii* | MZ520620 | MZ520623 |
|  | HKAS9613 | China | 0, I | *P. corensis* | OM746520 | OM746352 |
|  | BJFC-ZJ01 | China | 0, I | *P. armandii* | PP101607 | PP101617 |
|  | BJFC-ZJ02 | China | 0, I | *P. armandii* | PP101608 | PP101618 |
| *C. bethelii* | BCpC-15 | USA | 0, I | *P. contorta var. latifolia* | L76477 | - |
|  | BLvC-8 | USA | 0, I | *P. contorta var. latifolia* | L76478 | - |
| *C. castaneae* | HMAS18841 | China | II, III | *Castanea* sp. | OM746524 | OM746356 |
|  | HMAS8970 | USA | 0, I | *P. ponderosa* | OM746525 | OM746357 |
| *C. coleosporioides* | Ccol-yh3-FP | Canada | - | - | JN943207 | - |
| *C. comandrae* | MICH253364 | USA | II, III | *Com. umbellata* | MK193825 | MK208293 |
| *C. comptoniae* | UBC-F5871 | USA | II, III | *Comptonia peregrina* | OM746545 | OM746377 |
| *C. flaccidum* | FLAS-F-55559 | Finland | II, III | *Paeonia officinalis* | OM746551 | OM746383 |
|  | HMAS82720 | Russia | II, III | *Pae. lactiflora* | OM746561 | OM746393 |
| *C. floridanum* | MICH299992 | USA | 0, I | *P. palustris* | OM746576 | OM746408 |
| *C. fusiforme* | HMAS56356 | China | II, III | *Q. variabilis* | OM746583 | OM746415 |
|  | HMAS9043 | USA | II, III | *Q. emoryii* | OM746586 | OM746418 |

| **Species** | **Specimen No.** | **Country** | **Spore stage** | **Host** | **GenBank Accession No.** | |
| --- | --- | --- | --- | --- | --- | --- |
|  |  |  |  |  | **ITS** | **LSU** |
| *C. keteleeriae* | HMAS11129 | China | III | *Keteleeria davidiana* | - | OM746421 |
|  | HMAS638 | China | III | *K. davidiana* | - | OM746422 |
| *C. mongolicum* | HMAS242639 | China | I, III | *Q. mongolica* | OM746589 | OM746423 |
|  | ZP-R7 | China | II, III | *Q. mongolica* | OM746590 | OM746424 |
| *C. murrayanae* | MICH301494 | USA | 0, I | *P. murrayana* | OM746591 | OM746425 |
| *C. myricae* | MICH253485 | Canada | II, III | *Myrica asplenifolia* | OM746593 | OM746427 |
|  | MICH253505 | Canada | II, III | *M. gale* | OM746594 | OM746428 |
| *C. occidentale* | MICH253479 | USA | II, III | *R. gandfalii* | OM746595 | OM746429 |
|  | MICH253481 | USA | II, III | *R. aureum* | OM746597 | OM746431 |
| *C. orientale* | HMAS242640 | China | II, III | *Q. aquifolioides* | OM746598 | OM746432 |
|  | HMAS242641 | China | II, III | *Q. aquifolioides* | MK193820 | MK208291 |
|  | HMAS45784 | China | 0, I | *P. densata* | OM746606 | OM746440 |
|  | HMAS242500 | China | II, III | *Q. variabilis* | OM746604 | OM746438 |
|  | HMAS242501 | China | II, III | *Q. variabilis* | OM746605 | OM746439 |
|  | HMAS82717 | China | II, III | *Q. glandulifera* | MK193817 | MK208292 |
|  | BJFC-SX02 | China | II, III | *Quercus* sp. | PP195964 | PP195975 |
|  | BJFC-SX04 | China | II, III | *Quercus* sp. | [PP195965](https://www.ncbi.nlm.nih.gov/nuccore/?term=PP195964:PP195965%5baccn%5d) | [PP195976](https://www.ncbi.nlm.nih.gov/nuccore/?term=PP195975:PP195976%5baccn%5d) |
| *C. peridiatum* | NYBG267057 | USA | II, III | *Ribes* sp. | OM746612 | OM746446 |
|  | TSH-R14230 | Japan | II, III | *Pedicularis* sp. | OM746613 | OM746447 |
| *C. pini* | MD1 | Finland | 0, I | *Pinus* sp. | X83890 | - |
|  | GREEK1 | Greece | 0, I | *Pinus* sp. | X83908 | - |
| *C. pyriforme* | MICH253420 | USA | 0, I | *P. contorta* | OM746617 | OM746451 |
| *C. qinlingense* | HMAS56423 | China | II, III | *Q. aliena* | OM746619 | OM746453 |
|  | HMAS74356 | China | II, III | *Q. aliena* | OM746620 | OM746454 |
| *C. quercuum* | MICH253529 | Canada | II, III | *Q. rubra* | OM746621 | OM746455 |
|  | MICH253530 | Honduras | 0, I | *P. halepensis* | OM746622 | OM746456 |

| **Species** | | **Specimen No.** | **Country** | **Spore stage** | **Host** | **GenBank Accession No.** | | |
| --- | --- | --- | --- | --- | --- | --- | --- | --- |
|  |  |  |  |  |  | **ITS** | **LSU** |  |
| *C. ribicola* | ZP-R524 | China | II, III | *R. nigrum* | OM746631 | OM746465 |  |  |
|  | UBC-F5890 | Canada | II, III | *R. nigrum* | OM746644 | OM746478 |  |  |
| *C. ribis-taedae* | HMAS52871 | China | II, III | *R. nigrum* | OM746662 | OM746496 |  |  |
|  | FLAS-F-16581 | USA | 0, I | *P. taeda* | OM746664 | OM746498 |  |  |
| *C. strobilinum* | FLAS-F-53222 | USA | 0, I | *P. taeda* | MK193823 | MK208285 |  |  |
|  | CSt-2 | USA | 0, I | *P. elliotii* | L76482 | - |  |  |
| *Cronartium* sp. | HMAS49226 | USA | II, III | *R. aureum* | OM746666 | OM746500 |  |  |
|  | HMAS41544 | China | II, III | *Saussurea bullockii* | OM746667 | OM746501 |  |  |
| *Ravenelia macowaniana* | WM3485 | South Africa | - | *Vachellia karroo* | KP687429 | KP661594 |  |  |
| *Ravenelia evansii* | [PREM61005](https://www.ncbi.nlm.nih.gov/nuccore/KP687425.1) | South Africa | - | *Vachellia davyi* | KP687425 | MG945999 |  |  |
